# Neuropathic Pain Induced Alterations in the Opioidergic Modulation of a Descending Pain Facilitatory Area of the Brain

**DOI:** 10.3389/fncel.2019.00287

**Published:** 2019-06-28

**Authors:** Ana Rita Costa, Paulina Carvalho, Gunnar Flik, Steven P. Wilson, Carlos Reguenga, Isabel Martins, Isaura Tavares

**Affiliations:** ^1^Departamento de Biomedicina – Unidade de Biologia Experimental, Faculdade de Medicina da Universidade do Porto, Porto, Portugal; ^2^i3S – Instituto de Investigação e Inovação em Saúde, Universidade do Porto, Porto, Portugal; ^3^IBMC – Instituto de Biologia Molecular e Celular, Universidade do Porto, Porto, Portugal; ^4^Charles River Laboratories Den Bosch B.V., ’s-Hertogenbosch, Netherlands; ^5^Department of Pharmacology, Physiology and Neuroscience, University of South Carolina School of Medicine, Columbia, SC, United States

**Keywords:** opioids, μ opioid receptor, neuropathic pain, descending pain modulation, dorsal reticular nucleus

## Abstract

Opioids play a major role at descending pain modulation but the effects of neuropathic pain on the brain opioidergic system remain understudied. Since descending facilitation is enhanced during neuropathic pain, we studied the opioidergic modulation of the dorsal reticular nucleus (DRt), a medullary pain facilitatory area, in the spared nerve injury (SNI) model of neuropathic pain. We first performed a series of behavioral experiments in naïve-animals to establish the role of μ-opioid receptor (MOR) in the effects of endogenous and exogenous opioids at the DRt. Specifically, we showed that lentiviral-mediated MOR-knockdown at the DRt increased sensitivity to thermal and mechanical stimuli while the MOR agonist DAMGO induced the opposite effects. Additionally, we showed that MOR-knockdown and the pharmacological blockade of MOR by CTAP at the DRt decreased and inhibited, respectively, the analgesic effects of systemic morphine. Then, we performed *in vivo* microdialysis to measure enkephalin peptides in the DRt and evaluated MOR expression in the DRt at mRNA, protein and phosphorylated form levels by quantitative real-time PCR and immunohistochemistry, respectively. SNI-animals, compared to sham control, showed higher levels of enkephalin peptides, lower MOR-labeled cells without alterations in MOR mRNA levels, and higher phosphorylated MOR-labeled cells. Finally, we performed behavioral studies in SNI animals to determine the potency of systemic morphine and the effects of the pharmacologic and genetic manipulation of MOR at the DRt. We showed a reduced potency of the antiallodynic effects of systemic morphine in SNI-animals compared to the antinociceptive effects in sham animals. Increasing MOR-cells at the DRt of SNI-animals by lentiviral-mediated MOR-overexpression produced no effects on mechanical allodynia. DAMGO induced anti-allodynia only after MOR-overexpression. These results show that MOR inhibits DRt pain facilitatory actions and that this action contributes to the analgesic effects of systemic opioids. We further show that the inhibitory function of MOR is impaired during neuropathic pain. This is likely due to desensitization and degradation of MOR which are adaptations of the receptor that can be triggered by MOR phosphorylation. Skipping counter-regulatory pathways involved in MOR adaptations might restore the opioidergic inhibition at pain facilitatory areas.

## Introduction

Opioids are paramount in the control of descending pain modulatory areas (Fields, 2004; Ossipov et al., 2010), but the effects of chronic neuropathic pain on the opioidergic modulation of pain control centers of the brain remain understudied. This is especially relevant as the maintenance of neuropathic pain may rely on increased descending facilitation (Kovelowski et al., 2000; Bee and Dickenson, 2008; Sotgiu et al., 2008; Martins et al., 2010, 2015b). Insights from human and rodent studies provide evidence of alterations in the supraspinal opioid system in neuropathic pain conditions. Positron emission tomography studies in patients with peripheral and central neuropathic pain revealed reduced μ opioid receptor (MOR) availability in cortical brain areas involved in pain modulation, such as the insula and the striatum, and also in the periaqueductal gray (PAG) and the thalamus (Jones et al., 2004; Willoch et al., 2004; Maarrawi et al., 2007). A recent study performed in rats with peripheral neuropathic pain confirmed a reduced availability of MOR in the cortical areas referred above in human studies and showed that this was paralleled by reduced expression of MOR (Thompson et al., 2018). In the rat, neuropathic pain was further shown to induce MOR adaptations involved in desensitization, such as reduced MOR-mediated-G-protein activity in the thalamus and PAG (Hoot et al., 2011) and increased MOR phosphorylation at the striatum (Petraschka et al., 2007).

The dorsal reticular nucleus (DRt) plays a unique role in the facilitation of pain transmission (Lima and Almeida, 2002; Martins and Tavares, 2017). The DRt establishes reciprocal excitatory connections with the spinal dorsal horn, through which it is thought to amplify pain transmission (Lima and Almeida, 2002; Martins and Tavares, 2017). Descending facilitation from the DRt is enhanced during neuropathic pain (Martins et al., 2010, 2015b) and contributes to spinal sensitization during neuropathic pain (Sotgiu et al., 2008). The opioidergic system represents a key modulatory system at the DRt since it can directly and indirectly modulate the spinal-DRt-spinal reverberative pathway. Indeed, MOR is expressed both in spinally and non-spinally projecting neurons (Pinto et al., 2008b). Opioids act through direct inhibition of DRt spinally projecting neurons and also through disinhibition of enkephalinergic interneurons which receive input from GABAergic interneurons expressing MOR (Pinto et al., 2008a). We have previously shown that opioids inhibit DRt descending facilitation (Martins et al., 2008) and that in a model of chronic inflammatory pain there is a loss of inhibitory opioidergic tone, likely produced by decreased MOR expression, which results in enhanced descending pain facilitation (Pinto et al., 2008a). The impact of neuropathic pain on the opioidergic modulation of the DRt has never been explored. In this study we sought to study the effects of neuropathic pain on the opioidergic modulation of the DRt by using the spared nerve injury (SNI) model of neuropathic pain.

We first performed a series of behavioral experiments in naïve animals which consisted on the evaluation of the effects of the pharmacological and genetic manipulation of MOR at the DRt. We also evaluated in naïve animals the effects of genetic or pharmacological blockade of MOR at the DRt on the analgesic effects of systemic morphine. Then, we evaluated, at the DRt of sham and SNI animals, the extracellular levels of the methionine- (Met) and leucine- (Leu) enkephalin peptides by *in vivo* microdialysis, the mu-opioid receptor (MOR) expression at mRNA, protein and phosphorylated form levels by quantitative real-time PCR and immunohistochemistry, respectively. Finally, in SNI animals, we also performed a series of behavioral experiments to determine the potency of systemic morphine and the effects of the pharmacological and genetic manipulation of MOR at the DRt.

## Materials and Methods

### Animals

All procedures were approved by the Institutional Animal Care and Use Committee of the Faculty of Medicine of the University of Porto and were performed according to the ethical guidelines for pain investigation (Zimmermann, 1983). Male Wistar rats (Charles River colony, France) were maintained at 22 ± 2°C on a standard 12/12 h light/dark cycle with food and water available *ad libitum*. The animals were acclimated to the housing facility for at least 1 week before any treatment. All procedures were conducted during the light phase between 9:00 am and 5:00 pm. The subjective bias when allocating the animals to the experimental groups was minimized by arbitrarily housing the animals in pairs upon their arrival, then the animals were randomly picked from the cage for each procedure. No a priori power analysis was performed. The sample sizes were based on common practice of the research group where by default 6 animals per group are used in experiments, giving us approximately 90% power to detect large differences (2 standard deviations) between two groups, for continuous outcomes.

### Lentiviral Vector Construction

Three lentiviral vectors (LV) were used, a control vector expressing EGFP (LV-EGFP) and vectors designed to knockdown (LV-MOR-R) or overexpress (LV-MOR-F) MOR. Viral vector production was performed as previously described (Martins et al., 2015a). Briefly, to construct LV-MOR-R and LV-MOR-F, the cDNA for MOR was cloned into a lentivirus transfer vector, in antisense or sense orientation, respectively, relative to the human synapsin promoter (hSYN). This transfer vector also contains an encephalomyocarditis virus internal ribosome entry site (IRES) and the enhanced green fluorescent protein (EGFP). The virus was produced by transfection of human embryonic kidney 293T cells with the transfer vector, a packaging plasmid (pCMVΔR8.92), a plasmid encoding the rev protein (pRSV-Rev) and a plasmid encoding the vesicular stomatitis virus G glycoprotein (pMD.G). The vector LV-EGFP was constructed similarly, using a transfer vector with the hSYN promoter driving expression of EGPF in place of MOR cDNA. The titer of the vectors was determined by quantitative real-time PCR and all vectors were used at 5 × 10^6^ TU/μL. The LV were handled under biosafety level 2 containment and operating conditions according to Biosafety In Microbiological Biomedical Laboratories [BMBL] (2009). The animals injected with the LV were housed under ABSL2 conditions for 48 h, and then housed at ABSL1.

### Neuropathic Pain Induction

The SNI model of neuropathic pain was induced as described previously (Decosterd and Woolf, 2000) in rats weighing 210 to 220 g, under isoflurane anesthesia. These body weight are used to allow the animals to reach 285–315 g 2 weeks later, which is the ideal weight range for the stereotaxic surgeries. Briefly, the tibial and common peroneal components of the left sciatic nerve were carefully isolated, ligated and then sectioned. The sural nerve was maintained intact. Sham-operated animals were submitted to the same procedure except that no lesion was made. At the end of the procedure, the muscle and skin were sutured and the rats returned to their respective cages.

### Stereotaxic Surgeries

Rats (naïve or subjected to SNI or sham surgery 2 weeks earlier) weighing 285 to 315 g were deeply anesthetized with an i.p. mixture of ketamine hydrochloride (60 mg/Kg) and medetomidine (0.25 mg/Kg) and placed on a kopf frame for the injection of LV or cannula implantation into the left DRt. At the end of surgery, the animals received 0.9% NaCl (0.1 ml/kg, s.c.) for rehydration followed by atipamezole hydrochloride (0.5 g/Kg, s.c.) to revert the anesthesia.

#### Vector Injection

Stereotaxic injections were performed for the injection of LV into the left DRt in two rostrocaudal parts of the left DRt as previously described (Martins et al., 2015a). Naïve animals were injected with 0.6 μl per site of either LV-MOR-R or the control vector LV-EGFP. SNI-animals were injected with 1 μl per site of either LV-MOR-F or LV-EGFP. A higher volume (1 μl) of LV-MOR-F, compared to LV-MOR-R, was injected at the DRt, as in preliminary experiments this volume allowed a more efficient over-expression of MOR. Nonetheless, the injection of 1 μl induced the spreading of the vector to the non-injected (contralateral) DRt in some animals. In some animals, at the completion of the lentiviral injections, a guide cannula was implanted above the left DRt, as explained below, for the injection of DAMGO.

The effects of the manipulation of MOR expression by the LV were tested before and at 7 days after stereotaxic injections. At 7 days after injection, the hSYN was previously shown to be fully active (Marques-Lopes et al., 2012; Martins et al., 2015a).

#### Cannula Implantation

A guide cannula was implanted into the left DRt for microdialysis or pharmacological experiments following the coordinates and experimental procedures described previously (Martins et al., 2013, 2015b).

### Microdialysis Experiments

One week after stereotaxic surgery, the stylet of the guide cannula implanted in sham- and SNI-animals (*n* = 6 each) was replaced with a 2 mm open length microdialysis probe (molecular weight cutoff 45–50 kDa; Brainlink BV, Groningen, Netherlands). For stabilization purposes, the probe was perfused with Ringer’s solution (140.0 mM NaCl; 4.0 mM KCl; 1.2 mM CaCl_2_; 1.0 mM MgCl_2_) for 2 h at a flow rate of 2.0 μl/min. Two microdialysate samples were collected in 30 min intervals, for 1 h, into mini-vials already containing 20 μl of 0.02 M formic acid. At end of each collection, the samples were immediately placed on dry ice and stored at -80°C until analysis.

### Opioid Peptide Analysis

Met- and Leu-enkephalin were measured by HPLC with tandem mass spectrometry (API-5000). After collection, a mixture of BSA, ascorbic acid, acetic acid, and internal standard (Leu-Enkephalin^13^C_6_-^15^N was added to the microdialysate samples. Samples were injected by an autosampler onto a Phenomenex column (100 × 3.0 mm; 2.5 μm particle size). The gradient mobile phase contained different concentrations of acetonitrile, formic acid, and ultrapurified water, and was delivered through at a flow rate of 0.3 μl/min. Column effluent was diverted to the waste from *t* = 0–3.4 min to avoid source contamination. The quantification range was 0.5–500 pM.

### Behavioral Assessment

Mechanical and thermal sensitivity were assessed by the von Frey and hot-plate tests, respectively, after a 30 min daily habituation of the animals to the experimenter and testing apparatus, for 1 week.

The von Frey test was performed by placing the animals on an elevated transparent cage with a mesh wire bottom allowing the stimulation of the plantar surface of the left hind paw with calibrated von Frey monofilaments (Stoelting, United States) with logarithmically incremental stiffness. In naïve animals, we used a series of calibrated monofilaments ranging from 0.41 to 100 g. Testing started with the 2 g filament applied perpendicular to the plantar surface for 3 s. The weakest filament that elicited a response was taken as the withdrawal threshold. Each animal was tested twice at an interval of 3 to 5 min, each value obtained was logarithmic transformed and averaged. Withdrawal thresholds were determined using the Dixon up-and-down method (Chaplan et al., 1994). In SNI- animals, which typically develop hypersensitivity to mechanical stimuli on the injured paw (Decosterd and Woolf, 2000), the test was performed by stimulating the lateral plantar surface of the left, injured, hind paw as previously described (Tal and Bennett, 1994). Briefly, we used a series of calibrated monofilaments (Stoelting, United States), starting with the monofilament exerting the lowest force 0.008 g, in a sequence of increasing forces. The threshold was considered the lowest force that evoked a brisk withdrawal to one of five repetitive applications. Animals were also tested twice and each value was logarithmic transformed and averaged. The von Frey test we performed at the day before SNI induction or sham surgery and 14 days after surgery to confirm the development of mechanical hypersensitivity. Additionally, these animals were monitored for signs of sedation and locomotion impairments.

The hot-plate test was used to study thermal hyperalgesia in naïve animals. The test was performed on a hot-plate system (BIO-CHP Cold Hot Plate Test). A rectangular Plexiglas chamber (35 cm high) with a removable top was used to confine the rat to a 16.5 cm × 16.5 cm hot-plate surface. During the habituation period, the animals were placed on the plate set at 35°C for 15 min. On the testing day, the hot-plate was set with a surface temperature of 52°C. Nociceptive threshold was quantified as the latency (in seconds) to licking, retraction of the hind paw or jump after placement of the rat on the hot-plate. A 30 s cut-off was used to avoid tissue damage.

### Pharmacological Experiments

The MOR agonist (D-ALA2,N-ME-PHE4,GLY5-OL)-enkephalin acetate (DAMGO) and MOR antagonist D-Phe-Cys-Tyr-D-Trp-Arg-Thr-Pen-Thr-NH2 (CTAP), both obtained from Sigma-Aldrich (Portugal), were used to test the effects of MOR activation at the DRt of naïve and SNI animals. Morphine hydrochloride, generously provided by Dr. Paulo Cruz (Porto Military Hospital, Porto, Portugal), was administered s.c. All drugs were dissolved in saline.

Three sets of experiments were conducted in naïve animals. In the first set, 0.1 ng of DAMGO (*n* = 7) or saline (*n* = 6) were microinjected at the DRt. In the second set, 0.1 ng of DAMGO were microinjected at the DRt of animals previously injected with LV-MOR-R (*n* = 7) or LV-EGFP (*n* = 6). In third set, morphine (4 mg/Kg) was administered simultaneously with 0.33 μg of CTAP or saline at the DRt, saline s.c. alone or CTAP alone (*n* = 6 each). Two sets of experiments were conducted in SNI animals. In the first set, saline (*n* = 6) or DAMGO at 0.1 (*n* = 5), 1 (*n* = 6) or 10 ng (*n* = 7) were microinjected at the DRt. In the second set, 10 ng of DAMGO were microinjected at the DRt of animals previously injected with LV-MOR-F (*n* = 7) or LV-EGFP (*n* = 6).

DAMGO or CTAP were microinjected in a volume of 0.5 μl, infused over a period of 1 min, 1 week after cannula implantation and/or lentiviral injections, using a stainless steel needle protruding 3 mm beyond the cannula. The effects of the drugs were tested before and 15 min after injection, at their peak action (Hurley and Hammond, 2000, 2001). In morphine plus CTAP experiments, morphine was injected first followed by CTAP 15 min later and testing was performed 15 min after CTAP injection (i.e., 30 min after morphine, at its peak action (Erichsen et al., 2005). The doses of DAMGO and CTAP were determined based on previous studies performed at the DRt (Pinto et al., 2008a) and other supraspinal pain modulatory areas (Hurley and Hammond, 2000, 2001; Jongeling et al., 2009). The effects of the drugs were tested by the von Frey and hot-plate tests. The experimenter also monitored qualitatively by gross observations any behavioral changes (catatonia, agitation, ataxia, sedation), as well as levels of alertness throughout the period of testing. All tests were conducted by an experimenter blinded to the treatments.

### Morphine Dose-Response Experiments

To evaluate the effect of MOR knockdown at the DRt on the analgesic potency of systemic opioids, we used naïve animals injected 1 week earlier with LV-MOR-R (*n* = 7) or LV-EGFP (*n* = 6) at the DRt. To evaluate the impact of SNI on the analgesic potency of morphine, we compared the antinociceptive potency of morphine in sham animals with the antiallodynic potency of morphine in SNI animals (*n* = 5 each). Naïve or sham animals were tested by the hot-plate test. SNI animals were tested by the von Frey test. The animals were injected first saline s.c. followed by incrementing doses of morphine (0.1, 1, 4, and 10 mg/Kg; s.c). Each dose of morphine was administered every 30 min immediately after testing of the previous dose. Data was converted to percent maximum possible effect (%MPE), as explained below. Dose-response curves were plotted as %MPE vs. dose and fitted with non-linear regression (variable slope model) to determine ED50 values with 95% confidence intervals (GraphPad Prism v7).

### Tissue Preparation and Immunohistochemistry

The animals were deeply anesthetized with an overdose of sodium pentobarbital (150 mg/Kg i.p.) and perfused through the ascending aorta for perfusion with 200 mL of calcium-free Tyrode’s solution, followed by 800 mL of a fixative solution containing 4% paraformaldehyde in 0.1 M phosphate buffer, pH 7.2. The brainstem were removed, immersed in fixative for 4 h followed by 30% sucrose in 0.1 M phosphate-buffered saline (PBS) overnight, at 4°C, and sliced at 40 μm in a freezing microtome.

#### Immunohistochemical Detection of MOR

In a first experimental set, MOR expression was determined in sham- and SNI- animals (*n* = 6 each group) at 3 weeks after SNI induction or sham-surgery. In a second experimental set, MOR expression was determined 1 week after injection of: (i) LV-MOR-R (*n* = 5) or LV-EGFP (*n* = 6) at the DRt of naïve animals; or (ii) LV-MOR-F (*n* = 7) or LV-EGFP (*n* = 6) at the DRt of SNI animals. One in every fourth section encompassing the DRt was incubated for 2 h in a blocking solution containing 0.1 M glycine and 10% normal swine serum (NSS) in 0.1 M PBS containing 0.3% Triton X-100 (PBS-T) follow by an incubation for 48 h, at 4°C, in rabbit polyclonal antibody against MOR (ref: RA10104; Neuromics, United States), diluted at 1:1000 in PBS-T containing 2% NSS. After washing with PBS-T, the sections were incubated for 1 h in a swine biotinylated anti-rabbit serum diluted at 1:200 (Dako, Denmark) diluted in PBS-T containing 2% NSS. The sections were washed again and incubated for 1 h in PBS-T containing the avidin-biotin complex (1:200; Vector Laboratories, United States). After washing in 0.05 M Tris–HCl, pH 7.6, bound peroxidase was revealed using 0.0125% 3,3′ -diaminobenzidinetetrahydrochloride (DAB; Sigma-Aldrich, St. Louis, MO, United States) and 0.025% H_2_O_2_ in the same buffer. The sections were then dehydrated and mounted in Eukitt. Five sections encompassing the rostro-caudal extent of the DRt were randomly taken from each rat and the numbers of MOR-immunoreactive (IR) neurons were counted into the left and right DRt using the 20× objective by an experimenter blinded as to the experimental group. No differences were detected between the left and right side of the DRt for either SNI- or sham-animals (data not shown) from the first experimental set, therefore, left and right cell profile counts were summed in each tissue section from this experimental set. The DRt was delimitated in an additional set of immunoreacted sections counterstained with formol-thionin (Donovick, 1974) according to the atlas of Paxinos and Watson (1998). The specificity of the antibody anti-MOR was previously tested by blocking the antibody with a blocking peptide in immunohistochemistry and western blot analysis (Pinto et al., 2008b). We further tested antibody specificity by performing negative controls with omission of either the primary or the secondary antibodies which blocked all the immunostaining.

#### Immunohistochemical Detection of pMOR

The expression of pMOR was determined in sham- and SNI- animals (*n* = 6 each group) at 3 weeks after SNI induction or sham-surgery. One in every fourth section encompassing the DRt was processed for pMOR immunodetection, following the procedure described above; using a rabbit polyclonal antibody against MOR phosphorylated at serine 375 (Ser375) (Cell Signaling Technology, United States) diluted at 1:800 and incubated for 24 h at room temperature and 48 h at 4°C. Five sections encompassing the rostro-caudal extent of the DRt were randomly taken from each rat and the numbers of MOR-immunoreactive (IR) neurons were counted into the left and right DRt using the 20× objective by an experimenter blinded as to the experimental group. No differences were detected between the left and right side of the DRt (data not shown) therefore, left and right cell profile counts were summed in each tissue section. The specificity of the anti-pMOR was previously tested in agonist-induced phosphorylation assays in HEK293 cells expressing MOR (Schulz et al., 2004; Doll et al., 2011) or a Ser375MOR mutant (Chu et al., 2008) and by preadsorption of the pMOR antibody with an antigenic peptide in immunohistochemistry analysis (Gonzales et al., 2011). We performed additional negative controls by omission of either the primary or the secondary antibodies. No immunostaining was detected in the negative controls.

### Quantitative Real-Time PCR

Three weeks after SNI- or sham- surgery rats were deeply anesthetized with an overdose of sodium pentobarbital (150 mg/kg i.p.) and sacrificed by decapitation. The brains were harvested and immediately stored at -80°C. The medulla was cut into a frozen transverse block (1 mm in depth) from -5.60 to -4.68 mm relative to the Interaural line (Paxinos and Watson, 1998) from which the DRt (left and right sides) were dissected out using a tissue micropunch (Stoeling, Chicago, IL, United States). Total RNA from the DRt was extracted using the TRI Reagent (Sigma-Aldrich, Portugal) by following the manufacturer’s protocol and the RNA integrity verified by agarose gel electrophoresis. The first strand cDNA synthesis was prepared at 42°C during 1 h, from 0.5 μg of total RNA using 200 U of reverse transcriptase enzyme (Nzytech, Portugal) and 500 ng of oligo(dT)12–18 (Nzytech, Portugal). To assess for potential contaminants, a control containing all reagents except the reverse transcriptase enzyme was included for each sample. The expression levels of MOR mRNA were then quantified by the standard 2^∧^(–delta delta CT) method using a StepOnePlus Real-Time PCR system (Applied Biosystems, United States) and a SYBR green chemistry (SYBR Select master mix, Applied Biosystems, United States). The following intron-spanning primers 5′-GCCATCGGTCTGCCTGTAAT-3′ and 5′-GAGCAGGTTCTCCCAGTAC-3′ were designed to amplify exon 2 and 3 from the MOR-1 transcript. Normalization was performed by amplification of rat GAPDH using the primers 5′-GCATGGACTGTGGTCCTCAG-3′ and 5′-CCATCACCATCTTCCAGGAG-3′. The thermal cycling conditions included an initial denaturation step at 95°C for 10 min, followed by 40 cycles at 95°C for 10 s, 60°C for 20 s, and 72°C for 30 s. Melting curve analysis of every qPCR was conducted to ensure amplicon specificity. The results were presented as relative differences to sham MOR mRNA at the DRt.

### Histology

**FIGURE 1:**
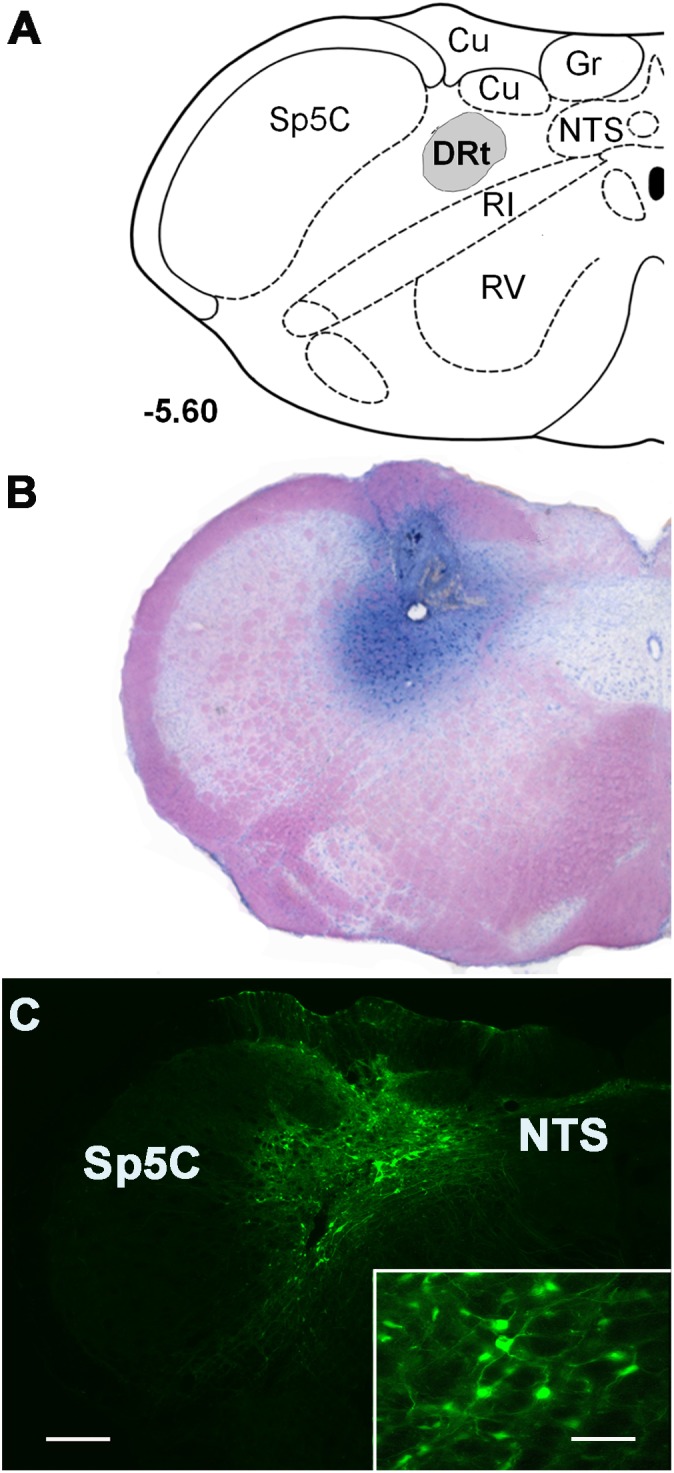
Representative injection sites at the DRt depicted in coronal sections. **(A)** Diagram depicting the location of the dorsal reticular nucleus (DRt), at 5.60 mm caudal to the interaural line [adapted from Paxinos and Watson (1998)]. **(B)** Representative photomicrograph of a thionin-stained section illustrating an injection site in the DRt identified by the needle tract surrounded by the dye staining. **(C)** Photomicrograph of a representative injection site of the control vector LV-EGFP injected at 0.6 μl into the DRt. The injection site includes a central area located within the DRt surrounded by EGFP-labeled neurons (better depicted at higher magnification in the insert). Scale bar in **C:** 200 μm (**B** is at the same magnification), scale bar of the enlargement: 50 μm. Abbreviations: Cu, cuneate nucleus; Gr, nucleus gracilis; IR, intermediate reticular nucleus; NTS, nucleus of the solitary tract; Sp5C, spinal trigeminal nucleus, pars caudalis; VR, ventral reticular nucleus.

Animals used in microdialysis experiments or injected with the LV were deeply anesthetized with an overdose of sodium pentobarbital (150 mg/Kg i.p.) and sacrificed by vascular perfusion as above. The animals used in pharmacological experiments were injected 0.5 μl of 0.6% Chicago sky blue dye (Sigma, United States) through the guide cannula, and sacrificed by decapitation. The brainstems were dissected out, and the medulla oblongata was coronally sectioned at 40 μm on a cryostat. Sections collected through the entire rostrocaudal extent of the DRt were stained by the formol-thionin (Donovick, 1974) for verification of probe location or blue dye injection (Figure 1A,B), as previously described (Martins et al., 2010, 2015a). In LV-EGFP-injected rats the injection site was observed by direct detection of EGFP labeling (Figure 1C). In LV-MOR-R- and LV-MOR-F-injected rats the location of the injection tract was observed in formol-thionin stained sections because the detection of EGFP was very faint. The EGFP transgene was inserted into the expression cassette in the second position of the bicistronic constructs which might be the raison why in the LV-MOR-F vector lower levels of EGFP expression were detected. In the LV-MOR-R vector, the lower levels of EGFP might be due to the RNA interference reaction induced by antisense RNA of MOR which also degrades EGFP RNA. Only animals with vector injections, cannula or probe placement centered in the DRt were included in data analysis.

### Calculation of MPE and Statistical Analysis

To enable the comparison of DAMGO or morphine effects after MOR knock down at the DRt (Figure 3, 4) and also the comparison of morphine effects in sham and SNI animals (Table 2), raw data was converted to percent maximum possible effect (%MPE) according to the equation: % MPE = (Post drug value-Pre-drug value)/(Ceiling value-Pre-drug value) × 100. In Figure 3, 4, “D7” (i.e., 7 days after LV injection) was taken as “Pre-drug value.” Paw withdrawal thresholds obtained in the von Frey test were log transformed for the estimation of MPE. To determine the %MPE from the data obtained in the von Frey test performed in naïve animals (Figure 3), the ceiling value (i.e., the maximum stimulus applied) was 100 g after DAMGO injection. To determine the %MPE from the data obtained in the von Frey test performed in SNI animals (Table 2), the ceiling value was the pre-operative (i.e., before SNI induction) value which was 15 g. To determine the %MPE from the data obtained in the hot-plate test, a cutoff latency of 30 s was taken as the ceiling value. %MPE values are presented as mean ± SD.

The behavioral effects of DAMGO, CTAP or the vectors, obtained in the hot-plate and von Frey test, and the %MPE of morphine in sham and SNI animals were analyzed by a two-way mixed ANOVA for repeated measurements. Mechanical threshold responses, obtained in the von Frey test, were logarithmic transformed to enable ANOVA analysis. In case of a significant interaction between group and time, we proceeded with pairwise comparisons using Tukey’s correction to adjust p-values for multiple testing. The effects of the LV on the number of MOR-IR cells in the left-injected (ipsilateral) and right (contralateral) DRt was analyzed by a two-way mixed ANOVA for repeated measurements (with the LV as a between factor and DRt sides as a within factor) followed by pairwise comparisons using Tukey’s correction. The unpaired *t*-test was used to compare the number of MOR- IR cells, pMOR-IR cells and MOR-mRNA levels between SNI- and sham-animals, the %MPE of DAMGO in LV-EGFP and LV-MOR-R and the ED50 of morphine in LV-EGFP and LV-MOR-R. The normality assumption was checked by inspection of the distribution of the variables both with q-q plots and histograms. However, we must acknowledge that the sample size limits the ability to detect departures from normality. The statistical analysis was performed by GraphPad Prism v7 and SPSS v24. The significance level was set at 0.05 and all statistical tests were two-tailed.

## Results

### MOR Expression at the DRt Produces Antinociception

The effects of MOR activation at the DRt of naïve animals were studied by using pharmacological and gene transfer approaches. The effects of both experimental approaches were tested by the von Frey and hot-plate tests.

**FIGURE 2:**
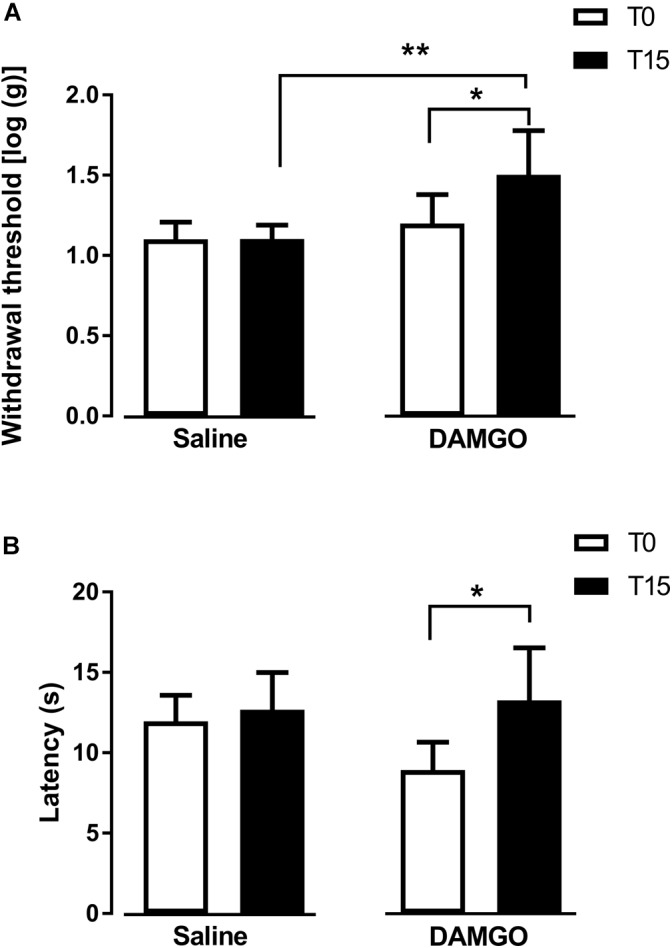
MOR activation at the DRt by the agonist DAMGO decreases mechanical and thermal sensitivity in naïve animals. Saline or DAMGO were injected at the DRt and their effects were assessed before (T0) and 15 min after (T15) injection by the von Frey **(A)** and the Hot plate **(B)** tests which evaluate mechanical and thermal sensitivity, respectively. Data are presented as mean ± SD (Saline *n* = 6; DAMGO *n* = 7). ^∗^*p* < 0.05, ^∗∗^*p* < 0.01.

In the first approach, we tested the effects of MOR activation by microinjection of DAMGO at 0.1 ng (*n* = 7) and saline (*n* = 6) into the left DRt. The analysis of the effects of DAMGO in the von Frey test revealed a significant interaction between treatment and time (*F*_1,11_ = 7.57, *p* = 0.019; Figure 2A). DAMGO increased withdrawal thresholds (1.5 ± 0.3) compared to before the injection (i.e., T0: 1.2 ± 0.2; *p* = 0.026) and saline (1.1 ± 0.1; *p* = 0.004; Figure 2A). Saline produced no significant effects (Figure 2A). Withdrawal thresholds before DAMGO and saline injections were not different (Figure 2A). The analysis of the effects of DAMGO in the hot-plate test revealed a significant interaction between treatment and time (*F*_1,11_ = 5.53, *p* = 0.038; Figure 2B). DAMGO increased latencies (13.3 ± 3.4 s) compared to before the injection (8.9 ± 1.9 s; *p* = 0.012; Figure 2B). Saline produced no significant effects (Figure 2B). Latencies before DAMGO and saline injections were not different (Figure 2B). No visible signs of sedation or ataxia were observed after DAMGO injection and the rats remained alert throughout the testing period.

**FIGURE 3:**
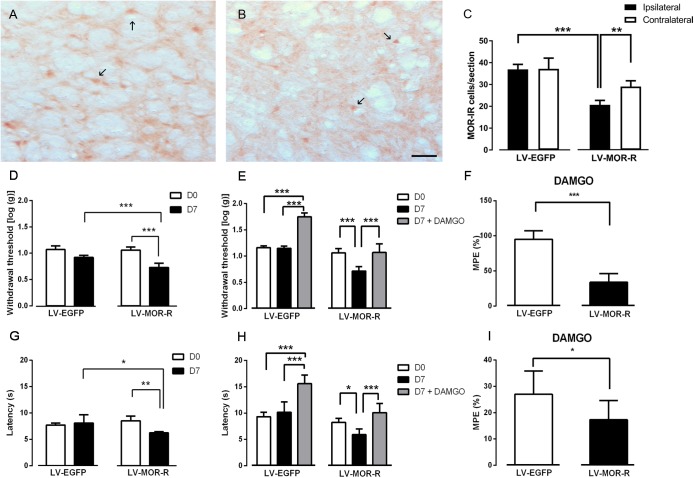
Lentiviral-mediated MOR knockdown at the DRt increases mechanical and thermal sensitivity in naïve animals. Representative photomicrographs of MOR-immunoreactive (IR) cells at the DRt of naïve animals injected with LV-EGFP **(A)** and LV-MOR-R **(B)**. Typical MOR immunolabeling is marked by arrows. Scale bar in **(B)**: 100 μm (A is at the same magnification). Data in **(C)** represents the number of MOR immunoreactive (IR) cells after lentiviral vectors injection into the DRt at the injected (ipsilateral) and contralateral side. LV-EGFP (*n* = 6) or LV-MOR-R (*n* = 5) were injected at the DRt and their effects were assessed before (D0) and 7 days (D7) after injection by the von Frey **(D)** and hot-plate **(G)** which evaluate mechanical and thermal sensitivity, respectively. An additional group of animals injected with LV-EGFP (*n* = 6) or LV-MOR-R (*n* = 7) into the DRt, was administrated 0,1 ng of DAMGO at the DRt. The effects of DAMGO were assessed before (D7) and 15 min after injection (D7+DAMGO) by the von Frey **(E)** and hot-plate **(H)**. Data in **(F)** and **(I)** represents the effects of DAMGO converted to percent maximum possible effect (%MPE) on the von Frey and hot-plate tests, respectively. Data are presented as mean ± SD.^∗^*p* < 0.05, ^∗∗^*p* < 0.01, ^∗∗∗^*p* < 0.001.

In the second approach, we used a lentiviral vector (LV-MOR-R) to knockdown the expression of MOR at the DRt. The effects were tested before (D0) and 7 days (D7) after DRt injections. The analysis of the numbers of MOR-IR neurons after the injection of LV-EGFP (*n* = 6) and LV-MOR-R (*n* = 5) at the left DRt revealed a significant interaction between vectors and DRt sides (*F*_1,9_ = 8.32, *p* = 0.018; Figure 3A–C). LV-MOR-R decreased the number of MOR-IR neurons in the left ipsilateral-injected DRt (20.7 ± 2.0) compared to the contralateral DRt (29.1 ± 2.7; *p* = 0.004) or compared to the ipsilateral LV-EGFP injected DRt (36.9 ± 2.3; *p* < 0.001; Figure 3C). The numbers of MOR-IR neurons were not different between the ipsilateral and contralateral DRt after LV-EGFP injection (Figure 3C). The analysis of the behavioral data obtained in the von Frey test revealed a significant interaction between vectors and time (*F*_1,9_ = 21.06, *p* = 0.001; Figure 3D). LV-MOR-R decreased withdrawal thresholds (0.7 ± 0.1) compared to before vector injection (i.e., D0: 1.1 ± 0.1; *p* < 0.001) and to LV-EGFP (0.9 ± 0.1; *p* < 0.001, Figure 3D). LV-EGFP also decreased withdrawal thresholds (0.9 ± 0.1) compared to D0 (1.1 ± 0.1; *p* = 0.004; Figure 3D). Withdrawal thresholds before LV-EGFP and LV-MOR-R injections were not different (Figure 3D). The analysis of the behavioral data obtained in the hot-plate test revealed a significant interaction between vectors and time (*F*_1,9_ = 11.21, *p* = 0.008; Figure 3G). LV-MOR-R decreased latencies (6.2 ± 0.2 s) compared to before vector injection (8.5 ± 0.8 s; *p* = 0.008) and to LV-EGFP (8.1 ± 1.6 s; *p* = 0.024, Figure 3G). LV-EGFP produced no significant effects (Figure 3G). Latencies before LV-EGFP and LV-MOR-R injections were not different (Figure 3G).

We also tested whether MOR knockdown at the DRt reduced the antinociceptive effects of the MOR agonist DAMGO injected at the DRt. We used a different set of animals simultaneously injected with LV-EGFP (*n* = 6) or LV-MOR-R (*n* = 7) and implanted with a guide cannula into the left DRt. One week later, we injected DAMGO at 0.1 ng, through the guide cannula. The effects were tested before (D0) and 7 days (D7; i.e., before the injection of DAMGO) after injection of the LV and 15 min after the injection of DAMGO (D7 + DAMGO). The analysis of the behavioral data obtained in the von Frey test revealed a significant interaction between vectors and time (*F*_2,22_ = 45.06, *p* = < 0.001; Figure 3E). In the LV-MOR-R group, DAMGO increased withdrawal thresholds (1.07 ± 0.2), compared to before the injection (0.7 ± 0.08; *p* < 0.001), to values similar to D0 (1.06 ± 0.08; Figure 3E). In the LV-EGFP group, DAMGO increased withdrawal thresholds (1.75 ± 0.07) compared to before the injection (1.15 ± 0.05; *p* < 0.001) and D0 (1.16 ± 0.04; *p* < 0.001; Figure 3E). The MPE of DAMGO in the von Frey test was lower in the LV-MOR-R group (33.8 ± 12.6%) compared to the LV-EGFP group (95.0 ± 12.3%; *p* < 0.001; Figure 3F). The analysis of the behavioral data obtained in the hot-plate test revealed a significant interaction between vectors and time (*F*_2,22_ = 11.47, *p* < 0.001; Figure 3H). In the LV-MOR-R group, DAMGO increased latencies (10.1 ± 1.8 s), compared to before the injection (5.9 ± 1.1 s; *p* = 0.04), to values similar to D0 (8.2 ± 0.7 s; Figure 3H). In the LV-EGFP group, DAMGO increased latencies (15.6 ± 1.6 s), compared to before the injection (10.2 ± 2.0 s; *p* < 0.001) and to D0 (9.3 ± 0.8 s; *p* < 0.001; Figure 3H). The MPE of DAMGO in the hot-plate test was marginally lower in the LV-MOR-R group (17.3 ± 7.4%) compared to the LV-EGFP group (27.0 ± 8.8%; *p* < 0.053; Figure 3I).

### MOR Expression at the DRt Contributes to the Analgesic Effects of Systemic Morphine

To evaluate whether the expression of MOR at the DRt of naïve animals is relevant for the analgesic effects of opioids administered systemically, we determined the effects of s.c. morphine after genetic MOR knockdown or pharmacological blockade of MOR at the DRt. The analgesic effects of morphine in both experimental approaches were tested by the hot-plate test.

**FIGURE 4:**
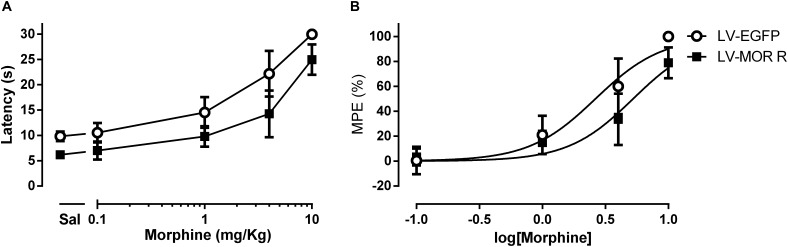
Lentiviral-mediated MOR knockdown at the DRt reduces the analgesic potency of systemic morphine in naïve animals. **(A)** Withdrawal latencies in the hot-plate test after saline administration followed by incrementing doses of morphine (0.1, 1, 4, and 10 mg/Kg) at 1 week after LV-EGFP or LV-MOR-R injection into the DRt. **(B)** Cumulative dose response curves of systemic morphine plotted as percentage of maximum possible effect (% MPE) and fitted by non-linear regression. The ED50 of morphine was 5.24 mg/Kg (95% CI: 4.24–6.48 mg/Kg) in the LV-MOR-R group and 2.64 mg/Kg (95% CI 1.97–3.53 mg/Kg) in the LV-EGFP group. Data are presented as mean ± SD (LV-EGFP, *n* = 6; LV-MOR-R, *n* = 7).

In the first experimental approach, we administered saline or morphine s.c. in a cumulative dosing procedure (0.1, 1, 4, and 10 mg/Kg) at 1 week after the injection of LV-EGFP (*n* = 6) or LV-MOR-R (*n* = 7) into the left DRt. The analysis of the behavioral data obtained in the hot-plate test revealed that morphine increased latencies in a concentration-dependent manner (*F*_4,44_ = 153.8, *p* < 0.001) and that latencies were lower in the LV-MOR-R group (*F*_1,11_ = 28,21, *p* < 0.001) consistently for all morphine doses, i.e., no interaction was detected between groups and morphine doses (*F*_4,44_ = 1.86, *p* = 0.135; Figure 4A). The morphine dose that produced 50% of the MPE, i.e., the ED50 in the LV-MOR-R group (ED50 = 5.24 mg/Kg (95% CI: 4.24–6.48 mg/Kg)) was 2-fold greater than in the LV-EGFP group (ED50 = 2.64 mg/Kg (95% CI 1.97–3.53 mg/Kg); *t*_11_ = 10.15; *p* < 0.001; Figure 4B) which indicates a reduction of the analgesic potency of morphine in the LV-MOR-R group.

**FIGURE 5:**
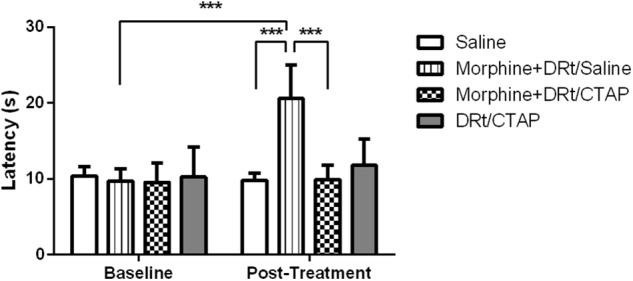
Pharmacological blockade of MOR at the DRt inhibits the analgesic effects of systemic morphine in naïve animals. Morphine was administered s.c. at 4 mg/Kg or saline in animals simultaneously injected with the MOR antagonist CTAP at the DRt. The animals were treated either with saline s.c. alone, morphine s.c. at 4 mg/Kg plus the injection of saline or 0.33 μg of the MOR antagonist CTAP at the DRt, or injected with 0.33 μg of CTAP alone at the DRt. Morphine was injected first followed by CTAP 15 min later. Withdrawal latencies were measured in hot-plate test before s.c. injections and 15 min after saline or CTAP injection at the DRt. Data are presented as mean ± SD (*n* = 6 each group). ^∗∗∗^*p* < 0.001.

In the second experimental approach, we determined the effects of morphine s.c. at 4 mg/Kg in animals simultaneously injected with the MOR antagonist CTAP at the DRt. The animals were treated either with saline s.c. alone (*n* = 6), morphine s.c. plus injection of saline or CTAP at the DRt (*n* = 6 each), or injected with CTAP alone at the DRt (*n* = 6). The analysis of the data obtained in the hot-plate test revealed a significant interaction between treatments and time (*F*_3,20_ = 21.52, *p* < 0.001; Figure 5). Morphine plus saline at the DRt increased latencies (20.6 ± 4.4 s) compared to baseline (9.7 ± 1.6 s) and to s.c. saline (9.8 ± 0.9 s; *p* < 0.001; Figure 5). The latencies of morphine plus CTAP at the DRt after treatment were not different from baseline (Figure 5). Morphine plus CTAP at the DRt (9.9 ± 1.9 s) significantly prevented the elevation of latencies induced by morphine plus saline at the DRt (*p* < 0.001; Figure 5). The injection of CTAP alone at the DRt produced no effects compared to baseline (Figure 5). Latencies at baseline were not different between the groups (Figure 5).

### Effects of SNI on Endogenous Opioid Peptide Levels and the Expression and Phosphorylation of MOR at the DRt

Extracellular levels of Met- and Leu-enkephalin at the DRt of sham- and SNI-animals (*n* = 6 each) were calculated by averaging the values of two consecutive DRt microdialysates obtained through the course of 1 h. In SNI-animals, an average of 4.20 ± 1.53 pM of Met-enkephalin and 1.17 ± 0.20 pM of Leu-enkephalin were measured in microdialysate samples (Table 1). Leu-enkephalin levels were below the limit of detection in two SNI-animals (Table 1). In sham-operated animals, Met- and Leu-enkephalin peptides could not be measured as they were below the limit of quantification.

**Table 1:** Met- and Leu-Enkephalin levels at the DRt of SNI animals.

	Met-Enkephalin (pM)	Leu-Enkephalin (pM)
Rat# 800	4.02	N.D.
Rat# 900	5.42	1.22
Rat# 1000	1.59	N.D.
Rat# 2200	3.53	0.89
Rat# 2900	5.73	1.17
Rat# 3000	4.94	1.39
**Mean ± SD**	**4.20 ± 1.53**	**1.17 ± 0.20**^a^

*^a^N.D., not detected – values below the lower limit of quantification. Mean calculated using values within the limit of quantification.*

**FIGURE 6:**
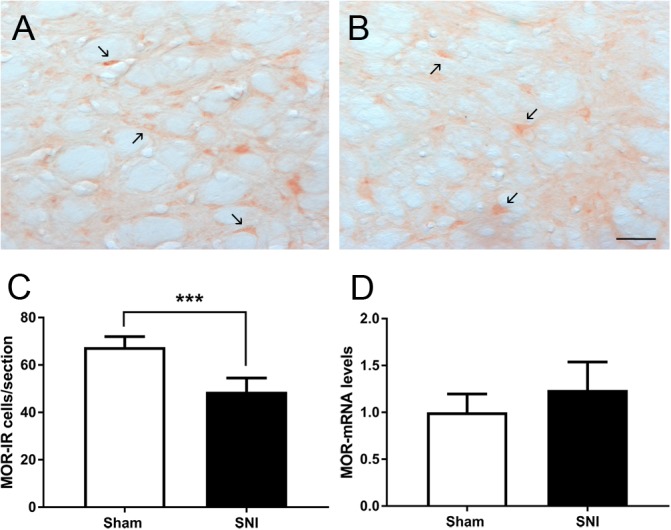
Effects of SNI induction on the expression of MOR at the DRt. Representative photomicrographs of MOR-immunoreactive (IR) cells at the DRt of sham- **(A)** and SNI- **(B)** animals. Typical MOR immunolabeling is marked by arrows. Scale bar in **(B)**: 100 μm (**A** is at the same magnification). Data in **(C)** represents the number of MOR-IR cells at the DRt of sham and SNI animals (*n* = 6/group). Data in **(D)** represents MOR mRNA levels at the DRt of sham (*n* = 3) and SNI (*n* = 5) animals, the results are presented as relative differences to sham MOR mRNA at the DRt. Data in C and D are presented as mean ± SD. ^∗∗∗^*p* < 0.001.

We studied the effects of SNI induction on the expression of MOR at the DRt by evaluating MOR mRNA levels and also the number of MOR-IR cells. The analysis of MOR mRNA levels at the DRt of sham (*n* = 3) and SNI (*n* = 5) animals revealed no significant differences between the two groups (*t*_6_ = 1.22; *p* = 0.268; Figure 6D). The analysis of the number of MOR-IR neurons at the DRt of sham- and SNI animals (*n* = 6 each) showed significantly lower numbers of MOR-IR neurons in SNI-animals (48.7 ± 5.8) compared to sham-animals (67.6 ± 4.3; *t*_10_ = 6.37; p < 0.001; Figure 6A–C).

**FIGURE 7:**
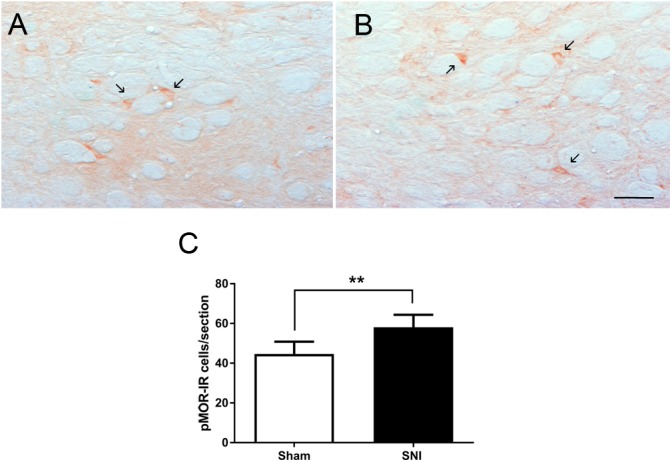
Neuropathic pain increases pMOR expression at the DRt. Representative photomicrographs of pMOR-immunoreactive (IR) cells at the DRt of sham- **(A)** and SNI- **(B)** animals. Typical pMOR immunolabeling is marked by arrows. Scale bar in **(B)**: 100 μm (A is at the same magnification). Data in **(C)** represents the number of pMOR-IR cells at the DRt of sham- and SNI- animals (*n* = 6/group). Data in **C** are presented as mean ± SD. ^∗∗^*p* < 0.01.

The effects of neuropathic pain on the phosphorylation of MOR was analyzed by evaluating the number of pMOR-IR at the DRt of sham- and SNI animals (*n* = 6 each). The analysis of the number of pMOR-IR cells showed significantly higher numbers of pMOR-IR cells in SNI-animals (58.1 ± 6.3) compared to sham-animals (44.6 ± 6.2; *t*_10_ = 3.71; *p* = 0.004; Figure 7A–C).

### The Antiallodynic Potency of Systemic Morphine in SNI Animals Is Reduced Compared to the Antinociceptive Effects in Naïve Animals

We evaluated the potency of systemic morphine in sham and SNI animals by the administration of morphine s.c. in a cumulative dosing procedure (0.1, 1, 4, and 10 mg/Kg). The effects of morphine were determined by the hot-plate test, in the sham group, and by the von Frey test, in the SNI group, and the results were converted in %MPE to enable the comparison. The MPEs of the antinociceptive and antiallodynic effects of morphine are reported in Table 2. The overall analysis revealed that the antinociceptive and antiallodynic MPEs in sham and SNI animals, respectively, increased in a concentration-dependent manner (morphine doses effect: *F*_3,24_ = 218.5, *p* < 0.001) and that the MPEs of morphine in SNI animals were lower than the MPEs of morphine in sham animals (group effect: *F*_1,8_ = 5.97, *p* = 0.040) for all doses tested as indicated by the absence of interaction between the MPEs of the groups and morphine doses (groups × morphine doses interaction: *F*_3,24_ = 0.50, *p* = 0.685; Table 2). The ED50 of the antiallodynic effect (ED50 = 3.7 mg/Kg (95% CI: 2.9–4.5 mg/Kg) was nearly 1.5-fold greater than the ED50 of the antinociceptive effect (ED50 = 2.5 mg/Kg (95% CI: 1.9–3.3 mg/Kg); *t*_8_ = 5.29; *p* < 0.001; Table 2) which indicates a reduced potency of morphine against SNI-induced pain behavior.

**Table 2:** Effects of morphine in sham and SNI animals.

Morphine (mg/Kg)	% MPE	
	Sham	SNI
0.1	7.2 ± 6.9	-6.6 ± 4.6
1	24.4 ± 9.7	11.2 ± 16.3
4	58.0 ± 13.4	51.7 ± 14.7
10	100 ± 0.0	93.4 ± 3.9
**ED50 mg/Kg (95%CI)**	**2.5 (1.9**-**3.3)**	**3.7 (2.9**-**4.5)**

*% MPE values are presented as mean ± SD.*

### SNI Induces an Impairment of MOR Function at the DRt

**FIGURE 8:**
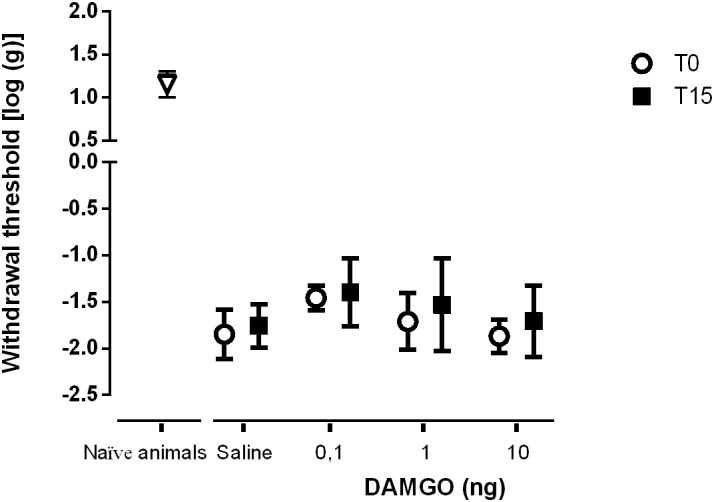
MOR activation at the DRt by the agonist DAMGO produces no effects on mechanical allodynia in SNI animals. Saline (*n* = 6) or DAMGO at 0.1 ng (*n* = 5), 1 ng (*n* = 6) or 10 ng (*n* = 7) were injected into the DRt and their effects were assessed before (T0) and 15 min after (T15) injection by the von Frey test which evaluates mechanical sensitivity. At T0 (i.e., 3 weeks after SNI induction), all SNI animals presented a marked mechanical hypersensitivity, indicative of mechanical allodynia, as shown by the decreased withdrawal thresholds compared to the withdrawal thresholds of the age-matched naïve animals (*n* = 13) used in Figure 2 for the injection of saline or DAMGO. The withdrawal thresholds of naïve animals correspond to the values obtained before the injection of saline or DAMGO in those animals. Data are presented as mean ± SD.

**FIGURE 9:**
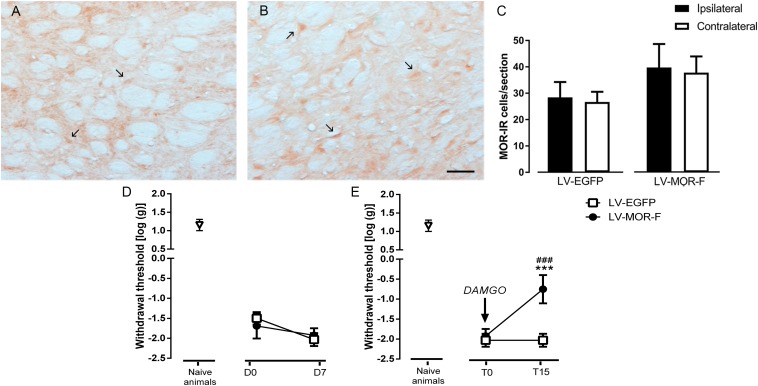
Effects of lentiviral-mediated MOR overexpression at the DRt on mechanical allodynia in SNI-animals. Representative photomicrographs of MOR-immunoreactive (IR) cells at the DRt of SNI animals injected with LV-EGFP **(A)** and LV-MOR-F **(B)**. Typical MOR immunolabeling is marked by arrows. Scale bar in **(B)**: 100 μm (A is at the same magnification). Data in **(C)** represent the number of MOR immunoreactive (IR) neurons after lentiviral vectors injection into the DRt at the injected (ipsilateral) and contralateral side. LV-EGFP (*n* = 6) or LV-MOR-F (*n* = 7) were injected at the DRt and their effects were assessed before (D0; i.e., 2 weeks after SNI induction) and 7 days (D7) after injection, by the von Frey test **(D)** which evaluates mechanical sensitivity. In an additional group of animals injected into the DRt with LV-EGFP (*n* = 6) or LV-MOR-F (*n* = 7), DAMGO at 10 ng was administrated at the DRt and its effects were assessed before (T0; i.e., 7 days after vectors injection) and 15 min (T15) after injection by the von Frey test **(E)**. At D0 (i.e., 2 weeks after SNI induction; **D**) and D7/T0 (i.e., 3 weeks after SNI induction; **D,E**), all SNI animals presented a marked mechanical hypersensitivity, indicative of mechanical allodynia, as shown by the decreased withdrawal thresholds compared to the withdrawal thresholds of the age-matched naïve animals (*n* = 13) used in Figure 2 for the injection of saline or DAMGO. The withdrawal thresholds of naïve animals correspond to the values obtained before the injection of saline or DAMGO in those animals. Data in **C,D,E** are presented as mean ± SD. ^∗∗∗^*p* < 0.001 vs. T0; ^###^*p* < 0.001 vs. LV-EGFP.

We performed two sets of experiments to determine the effects of SNI in MOR function at the DRt. In the first set of experiments we aimed at determining the effects of MOR activation. In the second set of experiments, since the number of MOR-IR cells was significantly decreased at the DRt of neuropathic animals, we aimed at testing the effects of restoring the number of MOR-IR cells by overexpressing MOR at the DRt. The effects of both approaches were tested by the von Frey test. Following SNI, the animals developed signs of mechanical allodynia in a manner similar to previous studies (Decosterd and Woolf, 2000; Martins et al., 2010), as shown in the von Frey test by the decrease of withdrawal threshold compared to age-matched naïve animals (Figure 8, 9). In the first approach, the effects of MOR activation were tested in SNI animals at 3 weeks after SNI induction by microinjection of saline (*n* = 6) or DAMGO at several doses [0.1 ng (*n* = 5), 1 ng (*n* = 6) and 10 ng (*n* = 7)] into the DRt. The overall analysis showed no effect of treatment (*F*_3,20_ = 2.34, *p* = 0.104), nor time (*F*_1,20_ = 3.16 *p* = 0.091) or interaction (treatment × time: *F*_3,20_ = 0.16, *p* = 0.922; Figure 8). No behavioral changes were detected after injection of each dose of DAMGO and the levels of alertness also remained unchanged after each injection.

In the second approach, to determine the effects of restoring the number of MOR-IR cells at the DRt of SNI animals, we used a lentiviral vector (LV-MOR-F) to overexpress MOR. LV-MOR-F (*n* = 7) or the control vector LV-EGFP (*n* = 6) were injected into the left DRt 2 weeks after SNI induction. The effects of the vectors were tested before (D0; i.e., at 2 weeks after SNI induction) and 7 days (D7) after injection, on the von Frey test. The analysis of the numbers of MOR-IR cells revealed that LV-MOR-F increased the number of MOR-IR cells regardless of DRt sides (*F*_1,11_ = 15.8, *p* = 0.002), no differences were found between the ipsilateral and contralateral DRt (*F*_1,11_ = 0.64, *p* = 0.439) nor a significant interaction (vectors × DRt sides *F*_1,11_ = 0.001, *p* = 0.973; Figure 9C). MOR-IR cells after LV-MOR injection (ipsilateral: 39.8 ± 8.9; contralateral: 37.8 ± 6.1) were higher compared to LV-EGFP injection (ipsilateral: 28.5 ± 5.8; contralateral: 26.7 ± 3.9; Figure 9C). The analysis of the behavioral data obtained in the von Frey test revealed only a significant effect of time (*F*_1,11_ = 22.02, *p* < 0.001), but no effect of vectors (*F*_1,11_ = 0.25, *p* = 0.629) nor a significant interaction (vectors × time: *F*_1,11_ = 3.28, *p* = 0.097; Figure 9D). Withdrawal thresholds at D7 (LV-EGFP: -2.0 ± 0.2; LV-MOR-F: -1.9 ± 0.2) dropped compared to D0 (LV-EGFP: -1.5 ± 0.2; LV-MOR-F: -1.7 ± 0.3; Figure 9D).

In order to confirm whether virally expressed MOR was functional, we used an additional set of animals that were simultaneously injected with LV-EGFP (*n* = 6) or LV-MOR-F (*n* = 7) and implanted with a guide cannula, at the left DRt, 2 weeks after SNI induction. Seven days later, we injected DAMGO at 10 ng, through the guide cannula, and tested its effects on the von Frey test. Overall, the analysis of the data revealed a significant interaction between vectors and time (*F*_1,11_ = 52.68, *p* < 0.001; Figure 9E). DAMGO increased withdrawal thresholds in LV-MOR-F-injected animals (-0.8 ± 0.4) compared to before the injection (-1.9 ± 0.2; *p* < 0.001) and compared to LV-EGFP (-2.0 ± 0.2; *p* < 0.001, Figure 9E). In contrast, the injection of DAMGO in LV-EGFP-injected animals produced no significant alterations (Figure 9E). Withdrawal thresholds before the injection of DAMGO were not significantly different between LV-EGFP- and LV-MOR-F-injected animals (Figure 9E).

## Discussion

We show, for the first time, the effects of neuropathic pain on the opioidergic modulation of the DRt, a major pain facilitatory area of the brain. Our main results indicate that MOR plays a key role in the analgesic effects of systemic opioids, which becomes impaired following SNI. Our results show that SNI increases extracellular-enkephalinergic peptides at the DRt, alongside with a reduction of the number of MOR-IR cells without alterations in MOR gene transcription. We further show that SNI increases the number of phosphorylated MOR-IR cells at the DRt. Given the involvement of MOR phosphorylation in the degradation and desensitization of the receptor, it is likely that the impairment of MOR in SNI-animals might be due this post-translational modifications of MOR. Taken together these alterations might contribute to a loss of inhibition of pain facilitation from the DRt which may underlie the imbalance of pain modulation toward pain facilitation during chronic pain and also impact on the efficacy of exogenous opioids in the treatment of neuropathic pain (Finnerup et al., 2015).

The present study shows that during SNI there is increased release of the endogenous opioid peptides Met- and Leu-enkephalin at the DRt. These peptides are likely released from local enkephalinergic interneurons and also from DRt afferent sources namely the RVM, the A_5_ noradrenergic cell group and the hypothalamus (Martins et al., 2008). Because these peptides were not detected in sham-animals we were not able to quantify the magnitude of this increase. This increase is consistent with the role of the endogenous opioids in the regulation of nociceptive transmission (Zubieta et al., 2001). Furthermore, a regional release of endogenous opioids has been shown in cortical and sub-cortical brain areas of patients with persistent pain of neuropathic origin (Jones et al., 1999; Willoch et al., 2004; Harris et al., 2007; Maarrawi et al., 2007). Studies performed in the rat with persistent pain of inflammatory and neuropathic origin showed up-regulation of Met-enkephalin at the spinal cord (Cesselin et al., 1980; Faccini et al., 1984; Noguchi et al., 1992; Sommer and Myers, 1995; Hossaini et al., 2014). In supraspinal pain modulatory areas, chronic inflammatory pain in the rat, increased enkephalin peptides at several brainstem nuclei including the PAG and RVM (Williams et al., 1995; Hurley and Hammond, 2001).

We found a reduction in the number of MOR-IR cells at the DRt of SNI-animals. This seems to be a common effect of neuropathic pain at pain modulatory areas. Neuropathic pain induced by peripheral nerve section reduces MOR immunostaining in the cell bodies of primary sensory neurons in DRGs and at their central terminal in the dorsal horn (deGroot et al., 1997; Goff et al., 1998; Porreca et al., 1998; Kohno et al., 2005; Sumizono et al., 2018) and also in cortical structures involved in pain modulation (Thompson et al., 2018). The reduction of the number of MOR-IR cells found in our study, contrary to the reduction of MOR immunostaining in primary sensory neurons (Kohno et al., 2005), is not because of down-regulation of MOR gene expression at the DRt since we found no alterations in MOR mRNA levels between sham- and SNI-animals. One possible explanation is that counter-regulatory adaptations may lead to increased traffic of MOR to degradative intracellular pathways. Indeed, MOR can be down-regulated by increased targeting to degradation in lysosomes (Law et al., 2000) which has also been observed to occur in a neuropathic pain model (Mousa et al., 2013). The phosphorylation of MOR is a post-translational modification which plays a major role in the regulation of MOR function after acute or prolonged exposure to agonists (Zhang et al., 2009; Williams et al., 2013; Allouche et al., 2014). An important mechanism triggered by MOR phosphorylation is the internalization of the receptor. Upon phosphorylation, MOR is internalized after what it can either be recycled back to the cell membrane or trafficked to lysosomes (Johnson et al., 2005). Here, we found increased pMOR-IR cells at the DRt of neuropathic animals. Furthermore, we detected MOR phosphorylation at the ser^375^ residue which represents a major phosphorylation site involved in MOR internalization (El Kouhen et al., 2001). Therefore, it is likely that the reduction of MOR-IR cells at the DRt of neuropathic animals could result from increased phosphorylation of MOR followed by internalization of the receptor and, ultimately, increased degradation in lysosomes.

The pharmacological and gene transfer studies in the DRt of naïve animals show that opioids modulate noxious thermal (heat) as well as and non-noxious mechanical (tactile) sensitivity through their actions at local MOR. The results are consistent with the activation of MOR resulting in inhibition of DRt facilitatory actions on both sensory modalities. Our results set for the first time a role for the DRt in the modulation of non-noxious mechanical sensitivity and the involvement of MOR in such actions. Additionally, the reduction of withdrawal thresholds to tactile stimuli, observed upon MOR knockdown, is indicative of the development of mechanical allodynia, i.e., a painful sensation caused by non-noxious mechanical stimuli. This increased mechanical sensitivity is likely due to decreased inhibition of tonic DRt descending facilitation. In line with this, it was shown that tactile allodynia is integrated predominantly at supraspinal brainstem nuclei (Saade et al., 2006), and the down-regulation of MOR at descending pain modulatory pain areas, induced by neonatal inflammation, was associated to the development of mechanical allodynia (Yan and Kentner, 2017). Additionally, tonic descending facilitation was shown to be involved in the mediation of mechanical allodynia after nerve injury (Ossipov et al., 2000).

To explore the effects of the alterations of the opioidergic system on MOR function at the DRt of SNI-animals, we determined the effects of the MOR agonist DAMGO on mechanical allodynia which is robustly developed after SNI induction. Additionally, mechanical allodynia constitutes a representative symptom of neuropathic pain in humans (Woolf and Mannion, 1999). We did not test thermal hyperalgesia, as changes in heat thresholds are difficult to measure in the SNI model (Decosterd and Woolf, 2000). We detected a decrease of the antinociceptive effects induced by DAMGO on mechanical allodynia, which is likely due to the reduction of MOR-IR cells at the DRt in neuropathic animals. Hence, based on the effects of MOR knockdown on mechanical sensitivity abovementioned, the reduction of MOR-IR cells at the DRt likely induces an impairment of the opioidergic inhibition of DRt descending facilitation in neuropathic animals. Incidentally, a reduction in the numbers of MOR-IR cells has also been shown in a model of chronic inflammatory pain, but this resulted in DAMGO-induced hyperalgesic effects at the DRt (Pinto et al., 2008a). This effect was likely caused by increased GABAergic input to the DRt, which is probably due to diminished opioidergic inhibition since local GABAergic interneurons express MOR, and GABA during inflammatory pain contributes to increasing descending pain facilitation (Martins et al., 2015a).

The loss of effect of DAMGO on mechanical allodynia suggests an impairment of the opioidergic inhibition of DRt descending facilitation in SNI-animals. Nonetheless, the impairment of MOR inhibitory actions cannot be solely explained by the reduction of MOR-IR cells since increasing the MOR protein at the DRt by lentiviral-mediated MOR gene expression did not alter mechanical allodynia. In these experiments, the MOR protein was efficiently up-regulated, as demonstrated by increased MOR-IR cells. Of note, the up-regulation of MOR was observed both at the ipsilateral (injected side) and contralateral side due to spreading of the vector which was injected at a higher volume than LV-MOR-R. MOR was also correctly trafficked and folded to the cell membrane, since microinjection of DAMGO at the DRt produced antiallodynic effects. However, it is worth noting that the effects of DAMGO in MOR-overexpression experiments were only partial since mechanical sensitivity did not revert to naïve thresholds, and the antiallodynic effects were obtained with a high dose of DAMGO. Therefore, based on the latter experiments with DAMGO together with the absence of effects of MOR up-regulation on mechanical allodynia, in spite of the high levels of endogenous enkephalin peptides, we suggest that neuropathic pain might also induce desensitization of MOR function at the DRt. The phosphorylation of MOR plays a major role in desensitization and the fact that MOR phosphorylation is increased at the DRt of neuropathic animals further argues in favor of this hypothesis. The effects of MOR knockdown on DAMGO effects in naïve animals, suggest that the remaining MOR at the DRt of naïve animals are still sensitive and therefore not phosphorylated, while, the absence of antiallodynic effects after MOR-overexpression in SNI-animals, further reinforces that MOR in SNI-animals might be highly subject to phosphorylation. The evaluation of pMOR levels after MOR knockdown and MOR-overexpression as well as the manipulation of MOR phosphorylation by increasing and decreasing the phosphorylation of MOR after MOR knockdown and MOR-overexpression, respectively, should confirm the role of MOR phosphorylation in MOR desensitization at the DRt. Phosphorylation induces desensitization of MOR by blocking the interaction of proteins with previously accessible regions of the receptor and changing the types of G protein the receptor interacts with and through which it mediates intracellular signaling (Johnson et al., 2005; Allouche et al., 2014). Increasing evidences of MOR desensitization induced by neuropathic pain include reduced MOR-mediated-G-protein activity in the thalamus and PAG (Hoot et al., 2011) and also increased MOR phosphorylation at the spinal dorsal horn (Narita et al., 2004) and the striatum (Petraschka et al., 2007). We propose that the mechanisms triggered by increased MOR phosphorylation, desensitization and increased targeting of MOR to degradation, could be due to prolonged activation of MOR by the high levels of endogenous opioid peptides found at the DRt of SNI-animals. Indeed, endogenous opioid peptide ligands, such as as enkephalins and endorphins induce robust desensitization and endocytosis (Llorente et al., 2012; Allouche et al., 2014) and the sustained release of endogenous peptides in the brain of neuropathic mice has been shown to induce desensitization of MOR and opioid tolerance (Petraschka et al., 2007).

In summary, the present study shows that induction of a model of neuropathic pain is associated with alterations in the opioidergic system at the DRt and that these alterations likely impact on downstream intracellular pathways that regulate MOR function. These alterations likely contribute to a loss of inhibition of pain facilitation from the DRt further enhancing descending facilitation during neuropathic pain. The treatment of neuropathic pain could benefit from the development of new compounds which can skip pathways involved in counter-regulatory mechanisms (Siuda et al., 2017).

## Ethics Statement

This study was carried out in accordance with the recommendations of the ethical guidelines of the International Association for the Study of Pain (Zimmermann, 1983). The protocol was approved by the Institutional Animal Care and Use Committee of the Faculty of Medicine of the University of Porto.

## Author Contributions

AC participated in the study design, performed stereotaxic surgeries for lentiviral injections and pharmacological experiments, immunohistochemical procedures and blinded cell counting of MOR and pMOR labeling, and manuscript drafting. PC performed microdialysis experiments. GF participated in the design of the microdialysis study, opioid peptide analysis, and writing of the manuscript. SW provided the viral vectors produced in his laboratory. CR participated in real-time PCR experiments and writing of the manuscript. IM and IT participated in the study design, discussion of the results, and writing of the manuscript. All authors have read and approved the final version of the manuscript finalized by IM.

## Conflict of Interest Statement

The authors declare that the research was conducted in the absence of any commercial or financial relationships that could be construed as a potential conflict of interest.
